# Treated and untreated cows housed side by side in tie-stalls and their respective risk of harboring *E*. *coli* resistant to antimicrobials

**DOI:** 10.1371/journal.pone.0310431

**Published:** 2024-11-07

**Authors:** Belinda Köchle, Véronique Bernier Gosselin, Heike Kaspar, Jens Becker

**Affiliations:** 1 Clinic for Ruminants, Vetsuisse-Faculty, University of Bern, Bern, Switzerland; 2 Department 505: Antibiotic Resistance Monitoring, Federal Office of Consumer Protection and Food Safety, Berlin, Germany; Michigan State University, UNITED STATES OF AMERICA

## Abstract

Parenteral antimicrobial treatment results in the excretion of antimicrobial-resistant bacteria. Dairy cows are commonly housed side by side in tie-stalls and often receive antimicrobial treatment. However, studies investigating treated cows as source of colonization of neighboring cows with resistant bacteria are scarce. Antimicrobial resistance (AMR) in cows (treated and untreated) in tie-stalls was investigated to assess their respective risks of carrying resistant bacteria. Furthermore, we analyzed associations of farm management with AMR. Case-control study: For isolation of indicator *Escherichia (E*.*) coli*, rectal swab samples were taken. Cows were sampled depending on treatment history and proximity to one another (cow A: recently treated parenterally; cow B: untreated, next to cow A; cow C: untreated, at considerable distance from all treated cows). Antimicrobial susceptibility was tested by microdilution. Associations of AMR with exposure to cow A, treatments, and management were analyzed using generalized mixed-effects logistic models. Susceptibility data on 571 isolates from 131 dairy farms were obtained. Almost no difference in proportions of resistant *E*. *coli* was observed between cows B and C (B: 53.4%; C: 57.2%; *P* = 0.52). Untreated cows had lower odds of carrying resistant *E*. *coli* than treated cows (B: OR 0.44, *P*<0.001; C: OR 0.54, *P* = 0.007). Non-pansusceptibility of isolates was associated with antimicrobial treatment (1 treatment: OR 2.11, *P* = 0.001; ≥2: OR 1.76, *P* = 0.043). Using manure on forage crops was associated with higher odds of pansusceptibility (OR 2.01, *P* = 0.004). For daily practice, with regard to the risk of AMR transmission, results of this study do not provide evidence for the need to separate treated cows from others during treatment in tie-stalls.

## Introduction

Antimicrobial use (AMU) in livestock farming contributes to the emergence and spread of antimicrobial resistance (AMR) [1]. Approximately 80% of the global production of antimicrobials is used in veterinary medicine [2]. There, 98.6% and 80% is attributed to food-producing animals in Europe and the U.S., respectively [1, 3]. The dairy sector, alongside swine and poultry production, has an elevated AMU particularly in low- and middle-income countries [4], where there is a concurrent increase in milk production and cow population. Developed countries on the other side, are experiencing a decline in production, number of dairy cows and farm counts [5]. Based on national sales data on antimicrobial drugs in the U.S., it can be inferred that this sector represented the least significant portion in terms of antimicrobial mass per route of drug administration [6]. In Switzerland, among all animal categories, dairy cows rank second (behind fattening cattle) with regard to the prescribed mass of active substance [7]. According to the same report, the most common reason for antimicrobial prescription in dairy cows is mastitis and mammary gland diseases (based on the number of treatments, not mass of active substance). Although a large proportion of dairy cows are housed in free stalls (notably due to free-stall barns typically housing a larger number of cows than tie-stall barns), tie-stalls still are a common housing system, representing 73% of dairy operations in Canada [8], and housing 40% of cows in Switzerland [9]. Rising levels of resistance and emergence of multidrug resistant pathogens have led governments and international organizations to take measures [10–13]. *Escherichia (E*.*) coli* is used as sentinel organism in resistance monitoring programs [12, 14, 15], since it is ubiquitous in a wide range of hosts and has a remarkable capacity to acquire resistance [15, 16]. Several studies analyzed the effect of antimicrobial treatment on *E*. *coli* susceptibility in dairy cows [17, 18] and other cattle production systems [19–21]. In calves, the carriage of florfenicol-resistant *E*. *coli* increased when housed in pens adjacent to that of florfenicol-treated calves (with physical contact), compared to calves housed in distant pens [22]. However, whether parenteral antimicrobial treatment in tied cows is associated with increased prevalence of AMR in untreated neighboring cows, which would suggest transmission, remains largely unexplored. The occurrence of AMR has also been linked to farm management practices [23–25], offering additional starting points for AMR mitigation.

The aims of the present study were to: (i) investigate the resistance of *E*. *coli* in untreated cows to evaluate physical proximity to treated cows as a risk factor for carrying resistant bacteria, (ii) investigate associations of AMU with AMR at the cow level, and (iii) investigate farm-level associations between management practices and AMR.

## Materials and methods

### Study design, sample size calculation, and ethical approval

This study was conducted in the frame of a study where associations of antimicrobial use with management practices and farm characteristics were investigated [26]. The study designs were a prospective case-control study regarding aim (i), and prospective non-randomized observational analyses regarding aims (ii) and (iii). Sample size calculation for the primary aim was conducted using R Studio version 4.2.0. (https://www.r-project.org/, Vienna, Austria, Ime4 package) using following assumptions: number of antimicrobial drugs to which indicator *E*. *coli* exhibit resistance: 2±2, difference to be detected: 1 drug, power: 80%, confidence interval: 95% [27]. A total of 126 observations per group were needed. Ethical approval for animal use was obtained from the competent authority (authorization no. BE76/2021). All participating farmers provided written informed consent. Sample sizes for aims (ii) and (iii) were the available populations from the aforementioned study [26].

### Farm recruitment

Recruitment took place between October 2021 and January 2023. Several articles were published in the agricultural press. Additionally, a short note was published on the webpages of the Swissherdbook breeding association and the Swiss tie-stall association. An invitation letter was sent to members of the Swiss tie-stall association (approximately 2200 members). Young farmers were recruited in different agricultural schools across Switzerland. Recruitment also occurred through word-of-mouth from enrolled producers. Study registration was facilitated through a webpage created specifically for the research project and also by contacting the first author via telephone or email. Participation was voluntary and not remunerated.

### Inclusion criteria and sample collection

To be included in the study, farmers needed to be milk producers and to keep cows in tie-stalls with each cow fixed to an individual stand. Sampled farms were located across Switzerland (19/26 cantons). Sampling was conducted during winter only, when cows were stabled the vast majority of time. Swiss tie-stalls implementing increased welfare standards must provide cows with short outdoor access on at least 13 days per month during winter [28] In summer, cows housed under these standards have access to pasture for a minimum of 26 days per month [28]. Hence, due to the decreased amount of exclusive contact between neighboring cows during summer, the months of June-September were excluded. Farm visits took place after notification by the farmers that a cow had received a parenteral antimicrobial treatment. Three different cows (cows A, B and C; i.e., one triplet) were non-randomly selected according to their treatment history and location: one treated cow (cow A), one of her immediate neighboring cows (cow B), and a third cow (cow C) tied at a distance of at least three untreated cows (cows X), or opposite to cows A and B in larger herds with several rows of stand. Cow A was eligible if lactating and having received a parenteral antimicrobial treatment within 4–7 days prior to sampling. This timeframe was selected due to selection pressure on *E*. *coli* exerted by parenteral AMU being highest at that time [17–21]. If antimicrobials were administered through intramammary route at dry-off, cow A was eligible as of 1 week postpartum. If treated with parenteral antimicrobial treatment during dry-off, eligibility was as of 2 weeks postpartum. Cows B and C were lactating, at least 2 weeks postpartum, and free from any antimicrobial treatment for at least 4 weeks prior to sampling. Neighboring cows of cows B and C (cows X) were also free from any antimicrobial treatment for the past 4 weeks at least. All cows of one triplet were sampled during the same farm visit and more than one triplet per farm was allowed whenever eligibility criteria were fulfilled.

Swabbing was performed using sterile cotton swabs (Copan Transystem, Brescia, Italy; Meus S.r.l., Padova, Italy) inserted into the rectum and immediately placed into the transportation medium suitable for Enterobacteriaceae. Samples were transported in cooled conditions to accredited laboratories by courier or, in rare cases, by mail from remote farms for next-day delivery.

### Data assessment

Antimicrobial treatment modalities (e.g., drug, dose, administration dates, indication, among others) of cow A were recorded during farm visits. Swiss farmers are obliged to record AMU in a farm treatment journal. If necessary, entries were clarified with the farmer or/and treating veterinarian. Data were entered into Microsoft Excel® (version 2016, Microsoft Corporation, Redmond, WA, USA). Additionally, cow A’s weight was estimated by measuring the heart girth with a measuring tape, indicating the estimated weight for European dairy breeds [29]. To collect general herd and management characteristics, a detailed questionnaire-based interview was conducted with the farmer during farm visits. Answers were entered into a Microsoft Access® datasheet (version 2016, Microsoft Corporation, Redmond, WA, USA) and exported to Microsoft Excel® for further handling.

### Isolation and identification of *E*. *coli*

Within 24 hours of collection, the rectal swabs were removed from their transport tube and directly spread (without prior dilution) onto standard plates for selective isolation of *E*. *coli* depending on agar availability (BROLAC agar, Biolab, Budapest, Hungary; MacConkey, Thermo Fisher Scientific, Basel, Switzerland; Chromogenic coliform agar, Merck, Darmstadt, Germany). The first 294 samples (48.3%) were inoculated onto BROLAC agar. Due to changes in laboratories’ protocols, MacConkey agar was utilized for the following 33 samples (5.4%), then chromogenic coliform agar was used for the remaining 282 samples (46.3%). Samples from the same triplet were inoculated onto the same agar. The plates were incubated under aerobic conditions for 24 hours at 37°C. One single colony per plate was randomly selected and cultured on trypticase soy agar containing 5% sheep blood (Becton Dickinson, Franklin Lakes, NJ, USA) or Columbia agar containing 5% sheep blood (Becton Dickinson, Franklin Lakes, NJ, USA) at 37°C for 24 hours. Identification and confirmation of *E*. *coli* was performed using matrix-assisted laser desorption/ionization time-of-flight mass spectrometry (MALDI-TOF MS; Bruker, Bremen, Germany). Isolates were frozen in microtubes with glycerol solution (Microbank™, Pro-Lab diagnostics, Wirral, UK) at -20°C until transfer to the University of Bern for storage at -80°C.

### Antimicrobial susceptibility testing

Antimicrobial susceptibility testing (AST) was performed after thawing and incubating the isolates on Columbia blood agar at 37°C for 24 hours. The minimum inhibitory concentration (MIC) of antimicrobials was determined in cation-adjusted Mueller-Hinton broth using EUVSEC3 Sensititre™ commercial test plates (Thermo Fisher Scientific, Basel, Switzerland). The panel of drugs included 15 antimicrobials. Isolates were classified as resistant (R) or susceptible (S) according to clinical breakpoints for Enterobacteriales provided by the European Committee on Antimicrobial Susceptibility Testing (EUCAST) [30]. If not available for a drug, recommendations from the Clinical and Laboratory Standards Institute (CSLI) were followed [31]. Isolates were classified as ‘pansusceptible’ (susceptible to all tested drugs) or ‘non-pansusceptible’ when resistance to at least one tested drug was observed. Moreover, isolates were classified as ‘multidrug-resistant’ (MDR) if resistant to at least one drug from a minimum of three different antimicrobial classes [32].

### Estimating antimicrobial use

Estimations of AMU were calculated in two ways to provide a) a detailed overview of the use of specific antimicrobials and b) an answer to the research question as to whether AMU is associated with AMR at the animal level.

Correspondingly, first, estimations of AMU are presented as ‘days under antimicrobial treatment’ for each drug used. For this, calculation of the treatment incidence (TI) was conducted for parenteral treatments and based on Defined Daily Dose (DDD) standards proposed by the European Medicines Agency (EMA) [33, 34]. The amount of drug administered was measured in mg of the respective active substance. Amounts of active substance per ml of therapeutic product were extracted from the Swiss Compendium of Veterinary Medicinal Products [35]. For amounts given in international units, recalculation into mg was conducted based on references provided elsewhere [36]. Treatments containing two active substances were counted as two treatments. A modified formula adapted from Pucken et al. (2021) [37] was used for TI calculation as shown below:

$$TI=\frac{total\;amount\;of\;drug\;administred\;(mg)}{DDD\;(\frac{mg}{kg})*\;number\;of\;cows\;treated*standard\;weight\;(kg)}$$

The unit of TI is days under treatment and was calculated twice, using the EMA-defined standard weight for adult dairy cows (500 kg) [34], and the estimated actual weight.

Second, for each cow from which bacterial isolates had been obtained, the number of treatments received was calculated. This was done to assess whether the number of treatments is associated with the number of drugs the bacterial isolate was resistant to.

### Statistical analyses

Descriptive statistics were done using NCSS® (version 22.0.3, NCSS, LLC, Kaysville, UT, USA) and R Studio. Results of AST were matched with treatment data and a set of selected farm management predictor variables. The difference in prevalence of *E*. *coli* exhibiting resistance among cows B and C was calculated using a chi-square test with Yate’s continuity correction. Zero-variance predictors and predictors with very unequilibrated variance with the outcome were excluded, leading to a set of 28 analyzable predictor variables (S1 Table). Further statistical analyses were performed with R Studio. Based on the skewed distribution of the number of drugs to which isolates showed resistance, model outcomes were classified as binary (‘pansusceptible *E*. *coli*’ and ‘non-pansusceptible *E*. *coli*’). In the first model, associations between AMR and farm-level predictor variables were investigated using generalized mixed-effects logistic models with a random effect for ‘farm’. In the second model, associations of AMR with cow-level number of antimicrobial treatments were investigated, with random effects for ‘farm’ and ‘cow ID’. For both models, multicollinearity among predictors was excluded if phi>0.6. Only predictor variables associated with the outcome at *P*≤0.2 were offered to the model. A stepwise backward selection procedure was conducted and variables were removed only if estimates of other predictors did not change by ≥30%. Model fit was evaluated using AIC. The number of drugs to which an isolate was resistant was compared between cows having received 1 or ≥2 treatments before sampling using the Wilcoxon rank sum test.

## Results

### Sample collection

**Fig 1:**
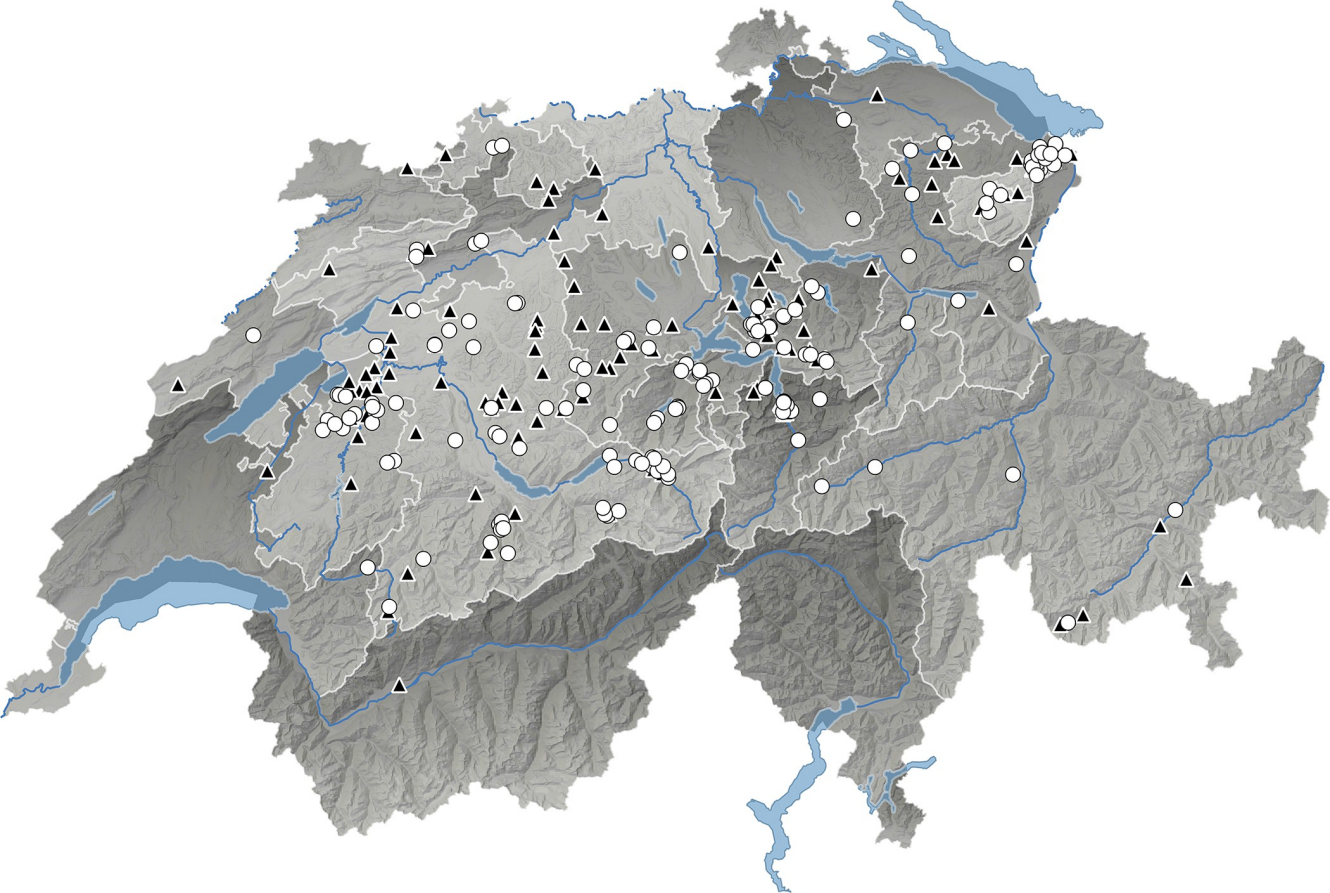
Map of Switzerland showing the geographical location of pre-enrolled farms (n = 280). Map source: QGIS®. Filled triangles (▲): sampled farms (n = 131). White dots (○): pre-enrolled farms without sampling (n = 149).

During the study period from November 2021 to April 2023, 280 farms were pre-enrolled to increase the probability of obtaining a sufficient number of samples (Fig 1). Sample collection took place over two winters (2021/2022, 2022/2023, October-May), depending on geographical location, altitude, and beginning of the grazing season. A total of 609 rectal swabs were taken from 584 cows on 131 of the 280 pre-enrolled herds, since not all farms contributed a suitable treated cow during the study period. Most farms contributed a single triplet (62.6%; 82/131), while others contributed two (23.7%; 31/131), or three or more triplets (13.7%; 18/131; range: 3–5). Most of the farms (51.9%; 68/131) contributed three isolates (35.7%; 204/571). Three single farms (2.3%; 3/131) contributed 2.6% (15/571), 2.5% (14/571) and 1.9% (11/571) of isolates, respectively. Eleven farms (8.4%; 11/131) contributed each nine isolates (17.3%; 99/571); two farms (1.5%; 2/131) contributed each eight isolates (2.8%; 16/571); one farm (0.8%; 1/131) contributed seven isolates (1.2%; 7/571); 22 farms (16.8%; 22/131) contributed each six isolates (23.1%; 132/571); eight farms (6.1%; 8/131) contributed each five isolates (7%; 40/571); two farms (1.5%; 2/131) contributed each four isolates (1.4%; 8/571); 11 farms (8.4%; 11/131) contributed each two isolates (3.9%; 22/571) and three farms (2.3%; 3/131) contributed only one isolate (0.5%; 3/571). From a total of 203 treated cows, four (2.0%) were sampled twice due to two treatment events during the study period.

### Farm characteristics and management

Mean herd size was 25 cows (±10.8; range 8–65) with an average annual milk yield per cow of 7,445 kg (±1,259; range: 3,000–11,500). The predominant breeds were Brown Swiss (46.6%; 61/131) and Holstein Friesian (27.5%; 36/131). Of all farmers, 91.6% (120/131) implemented welfare standards requiring regular outdoor access, typical for bovines in Switzerland [38]. The average cumulative time of outdoor access was 16.8 hours per month, i.e., 2.3% of a month’s time (±14.2 hours; range: 1.3–120). Nearly half of the farmers (49.6%; 65/131) applied manure on forage crops.

### Bacterial identification and antimicrobial susceptibility

No growth was observed on 34 plates (isolation rate of 94.4%), and 4 isolates were excluded due to the absence of pure culture after recovery from frozen storage. Therefore, a total of 571 *E*. *coli* isolates were available. 65.8% (376/571) of isolates were obtained from untreated cows and 34.2% (195/571) from treated cows (Table 1). A mean number of 0.8 drugs to which isolates exhibited resistance was observed among all cows (±1.0; range: 0–9). The mean number of resistances for cows A was 1.1 (±1.4; range: 0–9), and 0.7 (±0.7; range: 0–4) for cows B and C. The overall proportion of pansusceptible isolates was 39.6% (226/571). Almost no difference in the proportions of resistant *E*. *coli* was observed between isolates from cows B (53.4%; 101/189) and C (57.2%; 107/187, *P =* 0.52*)*. The proportion of resistant *E*. *coli* from cows A was 70.3% (137/195). Non-pansusceptible isolates exhibited resistance mostly to one antimicrobial drug (82.6%; 285/345), followed by two resistances (10.4%; 36/345) or more (7.0%; 24/345; range: 3–9). The overall proportion of MDR isolates was 4.2% (24/571). Results obtained from AST are presented in Table 2.

**Table 1:** Isolates obtained from untreated and treated cows stratified according to number of antimicrobial treatments received.

	Number of untreated cows (n = 406)	Number of treated cows (n = 203)	Number of cows undergoing one treatment event^1^ (n = 43)	Number of cows undergoing ≥2 treatment events^1^ (n = 160)
Number of isolates obtained	376	195	40	155

^1^ treatment events may comprise administration of one or ≥2 antimicrobial drugs

**Table 2:** Results of antimicrobial susceptibility testing for 571 *Escherichia coli* isolates obtained from rectal swabs in lactating cows on 131 Swiss tie-stall dairy farms collected during the winter periods of 2021/2022 and 2022/2023.

Antimicrobial drug	Antimicrobial class	Test range (mg/L)	Clinical breakpoints	Total number of resistant isolates	% resistant isolates among all tested isolates				% resistant isolates among all non-pansusceptible isolates
					Overall (Isolates n = 345)	Cow A (Isolates n = 137)	Cow B (Isolates n = 101)	Cow C (Isolates n = 107)	
Sulfamethoxazole	Sulfonamides	8–512	R ≥ 512^a^	334	58.5	69.7	50.8	54.5	96.8
Tetracycline	Tetracyclines	2–32	R ≥ 16^a^	52	9.1	13.8	6.9	6.4	15.1
Ampicillin	Penicillins	1–32	R ≥ 16^b^	27	4.7	8.7	4.2	1.1	7.8
Trimethoprim	Diamino- pyrimidines	0.25–16	R ≥ 8^b^	16	2.8	6.2	0.5	1.6	4.6
Chloramphenicol	Phenicols	8–64	R ≥ 32^a^	15	2.6	4.6	2.1	1.1	4.3
Gentamicin	Aminoglycosides	0.5–16	R ≥ 4^b^	7	1.2	3.6	0	0	2.0
Nalidixic Acid	Fluroquinolones	4–64	R ≥ 32^a^	5	0.9	2.6	0	0	1.4
Azithromycin	Macrolides	2–64	R ≥ 32^a^	3	0.5	1.5	0	0	0.9
Ciprofloxacin	Fluroquinolones	0.015–8	R ≥ 1^b^	2	0.4	1.0	0	0	0.6
Amikacin	Aminoglycosides	4–128	R ≥ 16^b^	2	0.4	0	0.5	0.5	0.6
Tigecycline	Glycylcyclines	0.25–8	R ≥ 1^b^	1	0.2	0	0	0.5	0.3
Colistin	Polymyxines	1–16	R ≥ 4^b^	0	0	0	0	0	0
Cefotaxime	3^rd^ generation Cephalosporines	0.25–4	R ≥ 4^b^	0	0	0	0	0	0
Ceftazidime		0.25–8	R ≥ 8^b^	0	0	0	0	0	0
Meropenem	Carbapenems	0.03–16	R ≥ 16^b^	0	0	0	0	0	0

^a^ Clinical and Laboratory Standards Institute (CSLI): M100, 32 ed. (2022), Clinical and Laboratory Standards institute, USA, 2022. https://EM100.Connect-CLSI M100 ED32:2022 (edaptivedocs.net).

^b^ The European Committee on Antimicrobial Susceptibility testing (EUCAST): Clinical Breakpoint Tables v. 13.0, 2023. https://EUCAST.Breakpoint.Tables.

### Treatment records for treated cows

A total of 300 parenteral treatments were recorded from the farm treatment journals. 57.3% included concurrent local antimicrobial therapy. The distribution of TI is displayed by standard and estimated actual weight separately (Table 3). Treatment incidences for each antimicrobial class were consistently higher with EMA standard weight than with the estimated actual weight. The most frequent reason for parenteral AMU was mastitis (45.0%), followed by reproductive disorders (14.3%), respiratory and digestive disorders (each 13.3%), musculoskeletal disorders (11.3%) and other indications (2.7%: fever of unknown origin, skin conditions, one case of eye enucleation).

**Table 3:** Estimated treatment incidences (TI) based on the standard weight for adult dairy cows defined by the European Medicines Agency and the measured weight of 203 treated cows, for each antimicrobial class used for parenteral treatments.

	TI (standard weight)				TI (estimated actual weight)			
Antimicrobial class	∑TI	mean	SD	Range	∑TI	mean	SD	Range
Penicillins	1699.1	11.9	14.9	0.9–112.6	1283.4	9.0	11.0	0.5–75.7
Aminoglycosides	254.8	8.0	9.6	0.2–34.1	193.0	6.0	7.3	0.1–21.3
Tetracyclines	142.5	2.8	1.9	0.8–9.8	118.6	2.3	1.8	0.6–10.4
Sulfonamides	87.5	2.8	4.2	0.1–17.1	70.0	2.3	3.7	0.1–17.2
Cephalosporines (3^rd^ and 4^th^ generation)^a^	36.4	6.1	2.5	2.5–9.0	23.9	4.0	1.5	1.8–5.6
Diamino-pyrimidines	32.2	1.8	0.7	0.7–3.4	24.9	1.4	0.7	0.5–3.4
Fluroquinolones^a^	17.0	2.1	1.5	0.6–4.8	12.7	1.6	1.2	0.4–4.1
Macrolides^a^	16.7	1.5	0.9	0.6–3.0	12.9	1.2	0.7	0.4–2.5

^a^ Highest priority critically important antimicrobials (HPCIAs) based on the importance to human medicine (WHO, 2018).

### Associations between AMR, treatments, and farm management

Results of the first and second models showed that AMR was associated with cows being treated and one farm management practice (Table 4), and with the number of treatments (Table 5), respectively. The odds of *E*. *coli* having ≥1 resistance were lower in untreated cows compared to treated cows (B: OR 0.44, *P*<0.001; C: OR 0.54, *P* = 0.007). Using manure on forage crops was associated with 2.01-fold higher odds of an isolate being pansusceptible (*P* = 0.004). Resistance was associated with both single and several treatments (1 treatment: OR 2.11, *P* = 0.001; ≥2 treatments: OR 1.76, *P* = 0.043). Cows with 1 treatment before sampling carried *E*. *coli* which exhibited resistance to 1.17±1.49 drugs (mean±sd), whereas cows with ≥2 treatments exhibited resistance to 1.01±1.21 drugs (Wilcoxon rank sum test; *P* = 0.708). Power analyses were performed using ‘R’ package *simr* using default setting for alpha = 0.05. Good power (i.e., probability that the test will reject the null hypothesis) was observed in both models (power for predictor 95% [confidence interval]: model in Table 4 (associations of AMR with various predictors) 93.00% [91.24, 94.50]; model in Table 5 (associations of non-susceptibility with treatments) 95.10% [93.57, 96.35]).

**Table 4:** Results of the logistic mixed regression model showing associations between AMR (non-pansusceptibility) and predictor variables, using data from 571 Escherichia coli isolated from rectal swabs of 584 cows from 131 tie-stall dairy farms in Switzerland.

Predictors	OR	CI (95%)	*P*
(Intercept)	1.81	1.19–2.77	0.006
Cow			
A (treated)	Referent		
B (untreated, located next to treated cow)	0.44	0.28–0.69	<0.001
C (untreated)	0.54	0.34–0.85	0.007
Applying manure on forage crops			
Yes	Referent		
No	2.01	1.25–3.22	0.004

**Table 5:** Results of the logistic mixed regression model on non-pansusceptibility in relation to number of treatments, using data from 571 *Escherichia coli* isolated from rectal swabs of 584 cows on 131 Swiss dairy farms.

Number of antimicrobial treatments		OR		CI (95%)		*P*
(Intercept)			2.31	1.30–4.10	0.004	
≥2	Referent					
1	1.21			0.60–2.44	0.598	
0	0.53			0.29–0.97	0.038	

## Discussion

From an AMR standpoint, separation during antimicrobial treatment does not appear necessary in tie-stall farms. The benefits of separating cows under treatment regarding the spread of infectious diseases or for animal welfare was beyond the scope of this study. If proximity to treated cows had been found to contribute to an increased prevalence of resistant *E*. *coli* in nearby untreated cows, the tightly controlled cow position within tie stalls would have permitted the identification of proximity to treated cows as risk factor for AMR. In our sampling frame, the exposure of cow B was defined as being located next to cow A, whereas cow C was not exposed. Following parenteral antimicrobial treatment, cows excrete resistant bacteria and antimicrobial residues (in feces and urine), which can contaminate their immediate environment. Excreted antimicrobial residues have been shown to contribute to maintenance of resistant bacteria in bedding [22, 39]. For cows adjacent to treated cows, exposure to resistant bacteria may result from contact with either the bedding or the treated cow (e.g., grooming). One possible explanation for our result may be a limited exposure attributable to the precise cow line-up, standing alongside each other (head to head and tail to tail). This may lead to a reduced probability of oral contact with feces or bedding material contaminated with resistant bacteria. Additionally, in the event of an untreated cow’s inoculation with resistant bacteria, it is possible that the dose was insufficient for these bacteria to successfully colonize and displace the dominant fecal flora and to become detectable by our bacterial isolation strategy. Indeed, a limitation of the present study was the characterization of a single isolate per sample. However, even when analyzing up to 10 isolates per cow, Singer et al. (2008) similarly reported no evidence of transmission of resistant *E*. *coli* strains to five untreated cows after antimicrobial treatment of five cows commingled among 150 cows in a free-stall barn [18]. In free stalls, cows may have a greater risk of exposure to feces from other cows as a result of not being tied, however a dilution effect (of resistant bacteria excreted by treated cows, by susceptible bacteria excreted by a larger number of untreated cows) may also take place. Berge et al. (2005) similarly reported no difference in fecal carriage of resistant *E*. *coli* in non-treated steers housed in pens containing a treated steer, compared to those in pens containing only non-treated steers (8–10 steers/pen) [19]. Although in the present study a large number of cow triplets from multiple farms were sampled, it cannot be excluded that transmitted resistant strains were missed due to the low sensitivity of *E*. *coli* isolation (i.e., no selective agar for drug-specific resistant strains was used) and low number of analyzed isolates relative to each cow’s fecal *E*. *coli* population.

We found that AMU was associated with AMR on the individual cow level. In studies investigating this association in cattle, such an association has been reported in dairy cow populations at the herd level [40, 41], and in dairy calves [20, 42, 43]. Other studies did not find such an association at the individual cow level [17, 18, 41]. The most common reason for systemic antimicrobial treatment in the treated cows in our study was mastitis (45%). Although the severity was not recorded, it is assumed that most of these were severe clinical mastitis cases [44]. Although severe mastitis usually is the least common form of clinical mastitis, the use of parenteral antimicrobials for the treatment of such cases is common in many countries [45, 46]. Unlike in the U.S., some antimicrobials are labeled in Switzerland for systemic use for the treatment of acute mastitis [47]. In an effort to reduce herd-level systemic AMU attributable to udder health, the implementation of evidence-based udder-health prevention strategies can be recommended, as it was previously shown to be successful [44].

The most commonly used antimicrobial class was penicillins (mean: 11.9 DDD/cow [standard weight]; 9 DDD/cow [estimated actual weight]). Penicillins dominated the antimicrobial sales market in livestock production across the majority of European countries in 2021 [3]. Kuipers et al. (2016) reported a tendency toward an increased use of penicillins in the Netherlands, which the authors attributed to the decline in the use of third-line antimicrobial drugs [45]. In line with the global efforts in reducing the use of certain important antimicrobials [48–50], high-priority critically important antimicrobials (HPCIAs) [51] in our study were employed at a very low incidence, suggesting overall compliance with national guidelines. An interesting observation was the difference in TI when conducting calculations with the EMA suggested standard weight and the estimated actual weight. Our results indicate that the average weight of dairy cows in Switzerland exceeds EMA suggestions, resulting in a lower TI among all antimicrobial classes due to the weight being taken into consideration in the denominator of the calculation formula. Studies demonstrated the utility of weight measurements, particularly the heart girth, as valuable indicators for estimating the weight of cattle [52, 53], with Pearson correlation values between the scale weight and measuring tape of 0.94–0.99 [52]. This suggests that estimations conducted using EMA standard weights should be interpreted with this caveat in mind.

Surprisingly, not fertilizing forage crops with cattle manure was associated with AMR in cows, with 2.01-fold higher odds of isolates being resistant. Unfortunately, neither the fate of manure produced on these farms, nor the alternative source of fertilizer were recorded. In most livestock farms, manure is collected in a pile or pit [54], before being used as fertilizer on crops. It is widely accepted that animal manure could act as a source of contamination of the environment with AMR genes [55–58] and drug residues [54, 57]. However, to our knowledge, there currently exists no evidence of AMR transmission back to the herd following manure application on fields used for forage production. The negative association observed in the present study might be explained by unmeasured confounding factors (e.g., specific handling of manure once stocked, other sources of fertilizer), and warrants further investigation.

Our study’s observational nature allows to establish associations between variables, not direct causality, which are nonetheless valuable to identify potential drivers of AMR. Selection bias may have been introduced due to the voluntary participation of farmers. Therefore, participants and their farms may not be representative of all Swiss tie-stall dairy producers.

In conclusion, this study did not provide any evidence for the need to separate neighboring cows over the course of antimicrobial treatments in tie stalls. The associations of isolate-level AMR with cow-level number of treatments and herd-level management practices point towards the general need to reduce AMU and to optimize farm management practices to mitigate the selection of resistant bacteria.

## Supporting information

S1 Table List of potential predictors for antimicrobial resistance used for statistical analyses. (PDF)
