# Supplementary material for: Treated and untreated cows housed side by side in tie-stalls and their respective risk of harboring E. coli resistant to antimicrobials
Source: PLoS One. 2024 Nov 7;19(11):e0310431. doi: 10.1371/journal.pone.0310431 (PMC11542856; doi:10.1371/journal.pone.0310431)
Supplement: S1 Table — (PDF) [file pone.0310431.s001.pdf]

Table S1. Potential predictors for antimicrobial resistance in 571 *Escherichia (E.) coli* at the farm level on 131 Swiss tie-stall dairy farms. Data on potential predictors were obtained through questionnaires conducted on-farm by the first author with the respective farmer. Antimicrobial resistance is investigated as *E. coli* being susceptible to 15 tested drugs or being non-susceptible.

[1]Number of cows, [2]annual thorough barn cleaning frequency, daily manure removal frequency, main manure removal system, main bedding, daily frequency of bedding refreshing, daily cleaning frequency of barn walkways, main cleaning system for barn walkways, main underground of barn walkways, daily cleaning frequency of the manger, [3]manger height, main manger system, annual high pressure cleaning frequency of the feeding alley, [4]use of hygienic powders on the lying area, [5]winter outdoor access, *daily cleaning frequency of the outdoor paddock, *[6]floortype of outdoor paddock, *[7]average number of outdoor access, [8]time spent outdoors, *[9]simultaneous outdoor access of all lactating cows, number of measures undertaken against flies, *[10]measures for rodent control, *[11]presence of birds in the barn, [12]presence of rodents in the barn, *usage of manure on forage crops, mountain pasturing of cows during summer, shared mountain pasturing with cows of other farms, [13]barn water origin

[1]including dry cows; [2]entire barn when animals are not present; [3]measured from laying area until upper rim; [4]chalk or other powders to reduce humidity; [5]on outdoor paddock, pasture, mix; [6]concrete, non-concrete; [7]in days per month; [8]in hours per day; [4,9,11,12] yes/no; [10]traps, poison, cat, mix, none; [13]source, community, mix.
*Univariate association with outcome at $P \leq 0.2$.
